# Supplementary material for: Extendable piezo/ferroelectricity in nonstoichiometric 2D transition metal dichalcogenides
Source: Nat Commun. 2023 Dec 20;14:8470. doi: 10.1038/s41467-023-44298-5 (PMC10733392; doi:10.1038/s41467-023-44298-5)
Supplement: Supplementary file 1 — Supplementary Information [file 41467_2023_44298_MOESM1_ESM.pdf]

Supplementary Information for

Extendable Piezo/ferroelectricity in Nonstoichiometric 2D Transition Metal Dichalcogenides

Yi Hu,[1,2] Lukas Rogée,[1] Weizhen Wang,[1] Lyuchao Zhuang,[1] Fangyi Shi,[1] Hui Dong,[1] Songhua Cai,[1] Beng Kang Tay,[2,3] Shu Ping Lau[1,*]

Affiliations:

[1] Department of Applied Physics, Hong Kong Polytechnic University, Hung Hom, Kowloon, Hong Kong, P. R. China.

[2] Centre for Micro- and Nano-Electronics (CMNE), School of Electrical and Electronic Engineering, Nanyang Technological University, Singapore 638798, Singapore.

[3] IRL 3288 CINTRA (CNRS-NTU-THALES Research Alliances), Nanyang Technological University, 637553, Singapore.

* Corresponding author. Email: apsplau@polyu.edu.hk (S.P.L.).

Supplementary Note 1. The theoretical basis for the preparation of nonstoichiometric two-dimensional (2D) compounds.

For simplicity, we assume that the used precursors are pure transition metals and pure chalcogen powders. In the calculation, M stands for a transition metal, X represents a chalcogen, V is a symbol of vacancy and M_i is a symbol of metal interstitial. Then, the concentrations of metal interstitial/intercalation defects, chalcogen vacancy defects, and metal vacancy defects as a function of chalcogen vapor pressure are calculated as follows[1,2]:

1.1 Metal interstitial/intercalation defect

Assuming that transition metal is completely ionized and then intercalated into TMDs layers.

$$\mathrm{M(g)} \rightleftharpoons \mathrm{M}_i^{\bullet\bullet\bullet\bullet} + 4e' \quad (1.11)$$

According to the law of mass equation.

$$K_1 = \frac{[\mathrm{M}_i^{\bullet\bullet\bullet\bullet}][\mathrm{e}']^4}{p_{\mathrm{M}}} \quad (1.12)$$

$$4[\mathrm{e}'] = [\mathrm{M}_i^{\bullet\bullet\bullet\bullet}] \quad (1.13)$$

Thus, the relationship between interstitial metal ion concentration and metal vapor pressure is:

$$[\mathrm{M}_i^{\bullet\bullet\bullet\bullet}] \propto p_{\mathrm{M}}^{1/5} \quad (1.14)$$

The chemical reaction of transition metal and chalcogen gives another relationship between metal vapor pressure and chalcogen vapor pressure.

$$\mathrm{M(g)} + 2\mathrm{X(g)} \rightleftharpoons \mathrm{MX_2(s)} \quad (1.15)$$

$$K_2 = [p_{\mathrm{M}}][p_{\mathrm{X}}]^2 \quad (1.16)$$

$$p_{\mathrm{M}} \propto p_{\mathrm{X}}^{-2} \quad (1.17)$$

By combining (1.14) and (1.17), interstitial metal concentration is achieved as a function of chalcogen vapor pressure.

$$[\mathrm{M}_i^{\bullet\bullet\bullet\bullet}] \propto p_{\mathrm{X}}^{-1/10} \quad (1.18)$$

Since transition metals may also be partially ionized, concentrations of interstitial metal with different valence state as a function of chalcogen vapor pressure can also be calculated as:

$$[\mathrm{M}_i^{\bullet\bullet\bullet}] \propto p_{\mathrm{X}}^{-1/8} \quad (1.19)$$

$$[\mathrm{M}_i^{\bullet\bullet}] \propto p_{\mathrm{X}}^{-1/6} \quad (1.20)$$

$$[\mathrm{M}_i^{\bullet}] \propto p_{\mathrm{X}}^{-1/4} \quad (1.21)$$

1.2 Chalcogen vacancy defect

If the TMDs MX_2 lose a chalcogen atom, a chalcogen vacancy is formed. Same calculations and speculations are as follows:

$$\mathrm{X_X} \rightleftharpoons \mathrm{V_X^{\bullet\bullet}} + 2\mathrm{e}' + \mathrm{X(g)} \quad (1.22)$$

$$2[\mathrm{e}'] = [\mathrm{V}_\mathrm{X}^{\bullet\bullet}] \tag{1.23}$$

$$K = \frac{[\mathrm{V}_\mathrm{X}^{\bullet\bullet}][p_\mathrm{X}][\mathrm{e}']^2}{[\mathrm{X}_\mathrm{X}]} \tag{1.24}$$

The concentration of chalcogen sites $[\mathrm{X}_\mathrm{X}]$ in the TMDs is normally unchanged. So, the final relation is:

$$[\mathrm{V}_\mathrm{X}^{\bullet\bullet}] \propto p_\mathrm{X}^{-3} \tag{1.25}$$

1.3 Metal vacancy defect

Due to the relatively low vapor pressure of transition metals, the formation of a metal vacancy in TMDs crystals usually costs two chalcogen atoms and requires four metals to compensate for the positive charges, thus the reaction formula is as follows:

$$4\mathrm{M}_\mathrm{M} + 2\mathrm{X}\,(\mathrm{g}) \rightleftharpoons \mathrm{V}_\mathrm{M}^{''''} + 4\mathrm{M}_\mathrm{M}^{\bullet} + 2\mathrm{X}_\mathrm{X} \tag{1.26}$$

$$\mathrm{M}_\mathrm{M}^{\bullet} \rightleftharpoons \mathrm{M}_\mathrm{M} + \mathrm{h}^{\bullet} \tag{1.27}$$

An equivalent formula can be obtained by combining formulas (1.26) and (1.27).

$$2\mathrm{X}\,(\mathrm{g}) \rightleftharpoons \mathrm{V}_\mathrm{M}^{''''} + 4\mathrm{h}^{\bullet} + 2\mathrm{X}_\mathrm{X} \tag{1.28}$$

$$K = \frac{[\mathrm{X}_\mathrm{X}]^2[\mathrm{V}_\mathrm{M}^{''''}][\mathrm{h}^{\bullet}]^4}{p_\mathrm{X}^2} \tag{1.29}$$

Based on (1.27), hole concentration $[\mathrm{h}^{\bullet}]$ equals four times the metal vacancy concentration $[\mathrm{V}_\mathrm{M}^{''''}]$. Thus, a final correlation between metal vacancy concentration and chalcogen vapor pressure can be expressed as:

$$[\mathrm{V}_\mathrm{M}^{''''}] \propto p_\mathrm{X}^{1/10} \tag{1.30}$$

Supplementary Note 2. Definition and calculation of effective piezoelectric coefficient d_{33} and $d_{31/32}$.

2.1 Definition of d_{33} and $d_{31/32}$

Piezoelectricity is electric polarization in a crystal caused by external stress. The piezoelectric effect can be described by $P_i = d_{ij}\sigma_j$, where P is polarization, d is a direct piezoelectric coefficient, σ is applied stress, and i and j imply polarization direction and stress direction. Piezoresponse force microscopy (PFM) is based on the inverse piezoelectric effect, where the applied voltage will induce sample deformation. The inverse piezoelectric effect can be expressed as $\varepsilon_j = d_{ij}E_i$, in which ε_j is the deformation produced by an electric field component E_i. In our experiment, drivel voltages were applied along the vertical direction while in-plane (IP) and out-of-plane (OOP) piezoelectric amplitude and phase were measured, resulting in a calculated piezoelectric coefficient of effective d_{33} and $d_{31/32}$. The so-called effective d_{33} and $d_{31/32}$ are because the measured value depends on the crystal topography, tip-sample contact condition, real space direction, and so on.

2.1 Calculation of effective d_{33} and $d_{31/32}$

A single frequency PFM mode was used to reveal IP piezoelectric response (Supplementary Figure 12), while dual AC resonance tracking (DART) PFM with lateral and vertical modes were used to measure IP and OOP ferroelectric domains, piezoelectric response and corresponding piezoelectric coefficient. In the DART OOP and IP modes, two frequencies near the center of the intrinsic resonance peak were chosen to collect two sets of amplitude and phase images (Supplementary Figure 3). However, those amplitudes are amplified by resonance rather than real/intrinsic amplitudes. The real/intrinsic amplitudes are then calculated by a simple harmonic oscillator model (SHO) (such as the bottom planes in Figure 3a and 3e). The grey spots indicate missing data which is unsuccessfully calculated during SHO calculation. The mean amplitude value and corresponding standard deviation for the interested region in the sample or substrate are exported by performing a Gaussian fit on the statistical diagram of all site values of the amplitude image. By linear fitting intrinsic amplitude to the drive AC voltage under consideration of the error bars, the mean value of effective piezoelectric coefficient (slope) and standard deviation are recorded (Figure 3b,f and Supplementary Figure 5,10,13). The value of the effective IP piezoelectric coefficient ($d_{31/32}$) is finally calibrated by a standard z-cut lithium niobate (LN) crystal.

A detailed region-cutting method was conducted to take account of the spatial variation of PFM amplitudes and evaluate the deviation of the piezoelectric coefficient in space. As illustrated in Supplementary Figure 5b,c, the intrinsic amplitude image of nanoflake in Supplementary Figure 5a acquired at a drive AC voltage of 4 V was divided into three regions, and the corresponding effective d_{33} was separately calculated through the method mentioned above. The calculated effective d_{33} values of area I, II and III were 0.61 ± 0.12 pm/V, 0.67 ± 0.14 pm/V and 0.63 ± 0.10 pm/V, respectively. The value differences between the three effective d_{33} are only in the second significant digit and are within the margin of standard error. Moreover, the average value of the effective d_{33} of three different regions is about 0.64 pm/V, which is very close to the effective d_{33} (0.65 ± 0.12 pm/V) counted from the total blue region (Supplementary

Figure 5a and Figure 3b). Although the local non-uniform distribution of amplitude signals will produce different effective d_{33}, the effective d_{33} calculated from the signal in the entire nanoflake region can be used to represent the piezoelectric coefficient of the nanoflakes.

Supplementary Note 3. The impacts of variation of chemical composition on the crystal structure and piezoelectric coefficient.

Defect/composition engineering was usually considered an effective/easy way to break the inversion symmetry of 2D materials to generate piezoelectricity and even spontaneous polarization. Thus, taking 2D $Cr_{1+\sigma}Se_2$ and $Ni_{1+\delta}Se_2$ nanoflake as examples, the σ/δ values of additional metal atoms were controlled by selenium vapor pressure/temperature and then determined by multi-point energy dispersive X-ray spectroscopy (EDS) (Supplementary Figure 7,9). The extra/intercalated metal atoms are verified by the positive value of σ/δ. Effective d_{33} of $Cr_{1+\sigma}Se_2$ nanoflake with the same thickness in different σ values were then determined by PFM characterization (Figure 3d and Supplementary Figure 8). Parallel experiments were also conducted on the $Ni_{1+\delta}Se_2$ systems (Supplementary Figure 9–11). As a result, both effective d_{33} of nonstoichiometric $Cr_{1+\sigma}Se_2$ and $Ni_{1+\delta}Se_2$ nanoflakes change with chemical composition (Figure 3d and Supplementary Figure 11).

The relatively simple nonstoichiometric $Cr_{1+\sigma}Se_2$ model is discussed first. The two terminals of the abscissa represent standard stoichiometric compounds of $CrSe_2$ and Cr_2Se_3, which are centrosymmetry with a theoretical effective d_{33} value of zero. The fitting curve after including the two endpoints depicts a mountain-like shape, indicating an evolution of first increase and then decrease of effective d_{33} as the σ value increases (Figure 3d). The changes in piezoelectric coefficient can be associated with the variation of generated electric dipole density during applying identical mechanical stress, where the dipole density is a vector field consisting of individual dipole vectors. Assuming that each intercalated metal atom can produce a dipole for $CrSe_2$, while each intercalated metal atom vacancy can similarly generate an equivalent dipole in the opposite direction for Cr_2Se_3. Thus, the effective d_{33} will almost linearly increase with the number of interstitial defects (increase in the σ value of $CrSe_2$) or vacancy defects (decrease in the σ value of Cr_2Se_3). However, the effective d_{33} value will decrease after reaching the peak value due to the electrical dipole in the opposite direction canceling out, thereby inhibiting the enhancement of the net dipole density. Similar results are also observed in $Ni_{1+\delta}Se_2$ nanoflakes (Supplementary Figure 11b), while $NiSe_2$, Ni_3Se_4 and NiSe are used as a zero-value coordinate point. It is worth noting that the amplitude of the dipoles formed in an interstitial ion defect or a vacancy defect is not strictly equal, leading to a shift of peak position in the actual curve.

Supplementary Note 4. Elaboration of IP PFM characterization and lithography.

Lateral PFM (IP mode) can detect an in-plane component of the dipoles as the lateral motion of the cantilever due to bias-induced surface shearing. Owing to the small contact area of the conductive tip on which drive AC voltage is applied, the revealed IP domains may not be as robust as the OOP one and the calculated effective piezoelectric coefficient is related to d_{31}/d_{32}. However, it is completely sufficient to qualitatively reveal the IP ferroelectric domains and calculate piezoelectric coefficients with reference to standard *z*-cut lithium niobate (LN) wafer. Moreover, angle-dependent IP PFM were performed to precisely ascertain the d_{31} and d_{32} component of IP piezoelectric response (Supplementary Figure 14).

Unlike switching the ferroelectric domain through patterns in the OOP lithography, two perpendicular scan lines with opposite voltages are employed to switch IP ferroelectric domains to avoid interaction between each scan line in the pattern (Figure 3j,k). Since the applied DC voltage is along the vertical direction, the electric field is a sphere centered on the tip. Besides, the diameter of the tip is very small, with tens of nanometers. Thus, the dwell time of each point in the line scan is prolonged compared with pattern lithography. After line scan lithography, the polarization directions on both sides of the scan line are theoretically opposite and perpendicular to the polarization direction on the scan line path. However, the IP PFM can only detect the polariton components perpendicular to the tip cantilever. Therefore, the phase patterning along the transverse line (black line) is obvious, while the phase along the longitudinal direction shows the same degree as the two side areas after lithography (white line) (Figure 3k). However, the horizontal polarization direction is obviously changed after longitudinal scanning (as shown by the point of intersection in Figure 3k), strongly proving the reverse of IP polarization of nonstoichiometric 2D TMDs. In addition, the phase and amplitude of the substrate are almost unchanged after line lithography, demonstrating that the phase change originates from the reversion of the IP polarization rather than the charge accumulation effect.

Supplementary Table 1. Summary of the space group of partial bulk 2D transition metal chalcogenides with different standard stoichiometric ratios. Space groups with centrosymmetry and non-centrosymmetry are marked in red and blue colors, respectively. It can be observed that most of the standard stoichiometric transition metal chalcogenides are centrosymmetric, and only a few of them exhibit non-centrosymmetry.

**Materials**	**Space group**	**Materials**	**Space group**
Cu_2S	*P*6$_3$/*mmc*<br>*P*2$_1$/*c*	CoS_2	*Pa*-3
CuS	*P*6$_3$/*mmc*	Co_4S_3	*F*-43*m*<br>*Fm*-3*m*<br>*P*6$_3$/*mmc*
CuS_2	*Pa*3	Co_3S_4	*Fd*-3*m*
Cu_9S_5	*R*-3*m*	V_5Se_8	*P*2/*m*
CrSe	*P*6$_3$/*mmc*	$FeTe_2$	*Pnnm*
Cr_2Se_3	*C*2/*m*<br>*P*-3*m*1<br>*R*-3	FeTe	*P*4/*nmm*<br>*P*6$_3$/*mmc*
MnSe	*Fm*-3*m*<br>*P*6$_3$/*mmc*<br>*F*-43*m*	NiSe	*P*6$_3$/*mmc*<br>*R*3*m*<br>*Cmcm*
$MnSe_2$	*Pa*-3	$NiSe_2$	*Pnnm*<br>*Pa*-3
		Ni_3Se_4	*C*2/*m*<br>*Fd*-3*m*

Supplementary Table 2. Effective d_{33} comparison between nonstoichiometric 2D $Cr_{1+\sigma}Se_2$ and $Ni_{1+\delta}Se_2$ nanoflakes and other 2D piezoelectrics. The intrinsic represents the inherent non-centrosymmetric structure with piezoelectricity, and the extrinsic represents no piezoelectric effect in perfect crystals and the piezoelectricity is obtained by breaking the central symmetry.

Materials	Origin of piezoelectric	Effective d_{33} (pm/V)	Thickness (nm)	Ref.
**SnS_2**	Extrinsic	2 ± 0.22	4	3
**MoO_2**	Extrinsic	0.56	8.7	4
**σ-Tellurene**	Intrinsic	1	2	5
**CdS**	Intrinsic	16.4	3.09	6
**σ-In_2Se_3**	Intrinsic	5.6	Bulk	7
		0.34	Monolayer	
**3R-MoS_2**	Intrinsic	≈ 0.9	18	8
**2H-$MoTe_2$**	Extrinsic	2.08	125	9
**MoS_2/WS_2**	Intrinsic	1.95 – 2.09	>1.5	10
**Janus MoSSe**	Intrinsic	0.1	0.7	11
**Doped graphene**	Extrinsic	1.4	Monolayer	12
**2D $Cr_{1+\sigma}Se_2$**	Extrinsic	0.65 ± 0.12	5.6	This work
**2D $Ni_{1+\delta}Se_2$**	Extrinsic	6.78 ± 0.6	47.8	This work

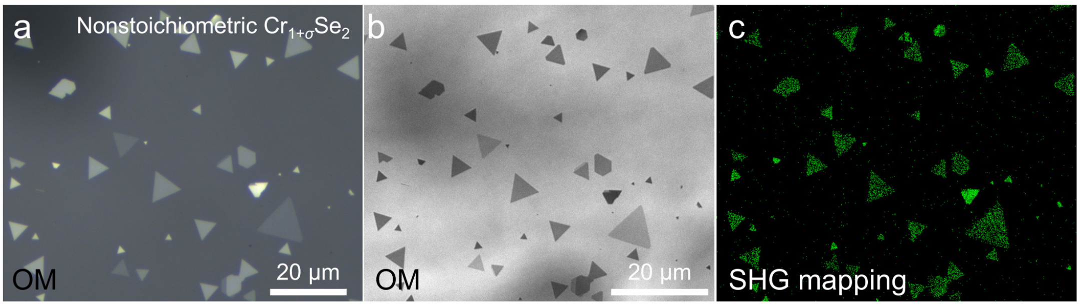

Supplementary Figure 1. Optical characterizations of nonstoichiometric 2D $Cr_{1+\sigma}Se_2$ nanoflakes. (a-b) Optical microscopy (OM) images and (c) second harmonic generation (SHG) mapping image of nonstoichiometric two-dimensional (2D) $Cr_{1+\sigma}Se_2$ triangular nanoflakes. As illustrated, nanoflakes in optical microscope images can correspond well to SHG mapping signals. Besides, there is no signal in the background, which proves that the SHG signal comes from the nanoflakes and confirms the broken symmetry of the nonstoichiometric 2D $Cr_{1+\sigma}Se_2$ triangular nanoflakes.

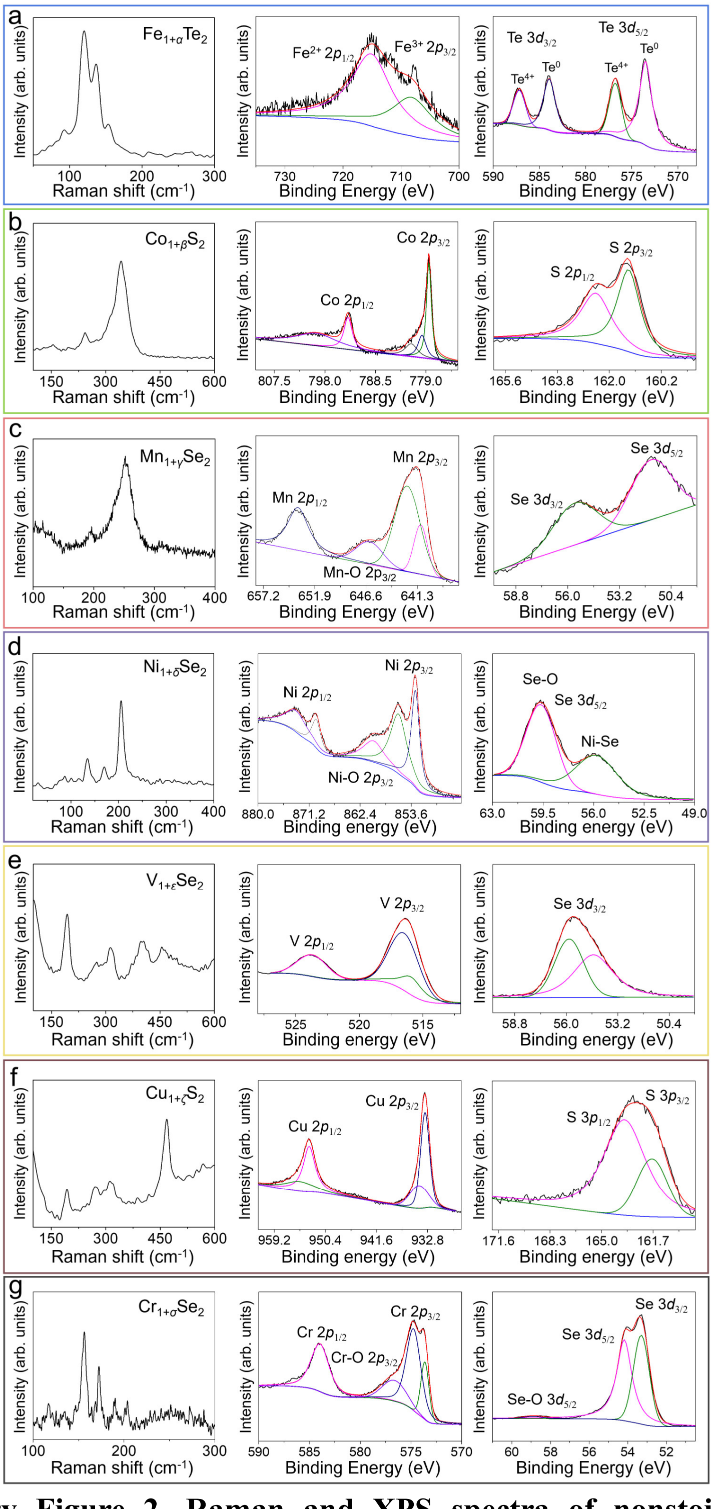

Supplementary Figure 2. Raman and XPS spectra of nonstoichiometric 2D nanoflakes. (a-g) Raman spectra (left panel), XPS core level spectra of metal 2*p* orbits (middle panel) and chalcogen inner-shell orbits (right panel) of $Fe_{1+\sigma}Te_2$ (a), $Co_{1+\beta}S_2$ (b), $Mn_{1+\gamma}Se_2$ (c), $Ni_{1+\delta}Se_2$ (d), $V_{1+\varepsilon}Se_2$ (e), $Cu_{1+\zeta}S_2$ (f), $Cr_{1+\sigma}Se_2$ (g). The XPS peaks of metals can only be perfectly fitted when there are multiple sub-peaks for fitting, indicating that there are indeed multiple chemical environments for metals.

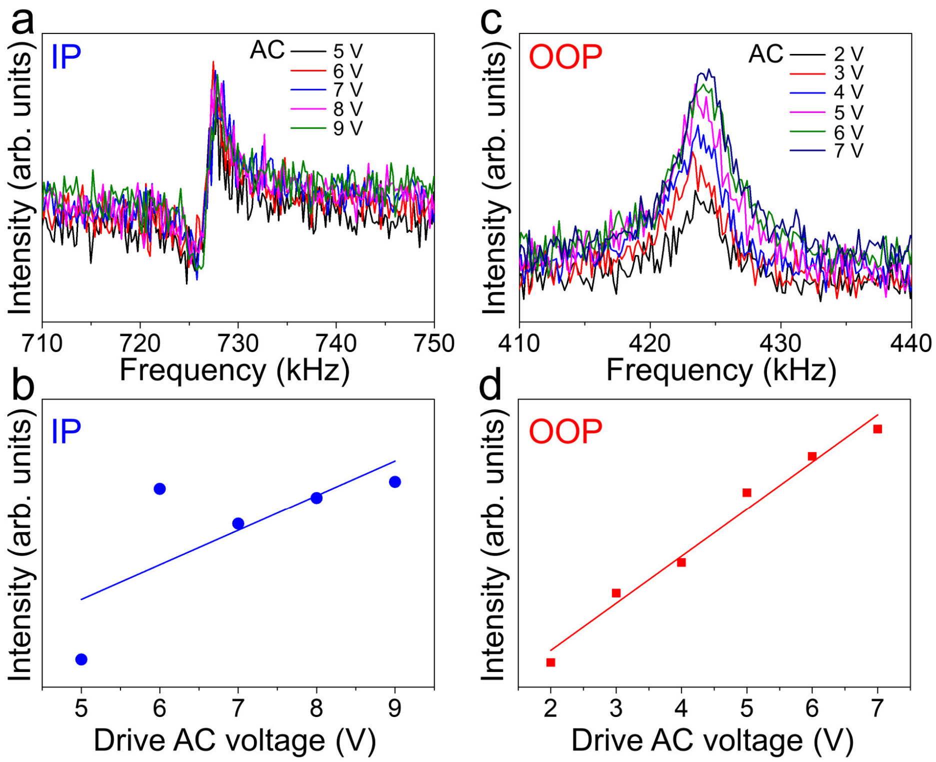

Supplementary Figure 3. PFM resonance curves of nonstoichiometric 2D $Cr_{1+\sigma}Se_2$ nanoflakes. (a) In-plane (IP) amplitude intensity *vs*. drive frequency curves under different drive AC voltages (as indicated by different color lines). AC indicates an alternating current. (b) IP resonance peak intensity as a function of drive voltages with raw data points (blue dots) and linearly fitted line. (c) Out-of-plane (OOP) amplitude intensity *vs*. drive frequency curves under different drive voltages as indicated by different colors. (d) OOP resonance peak intensity as a function of drive voltages. Blue spots, red frames and lines in (b) and (d) indicate raw data points and linearly fitted curves. The IP piezoelectric effect is insignificant, but an enhanced tendency of the amplitude with the increase of voltage can still be observed. OOP piezoelectric effects are evident and fit well. Since the resonance peak is not smooth, the intensity values of a fixed voltage at the resonance peak were read for voltage intensity fitting.

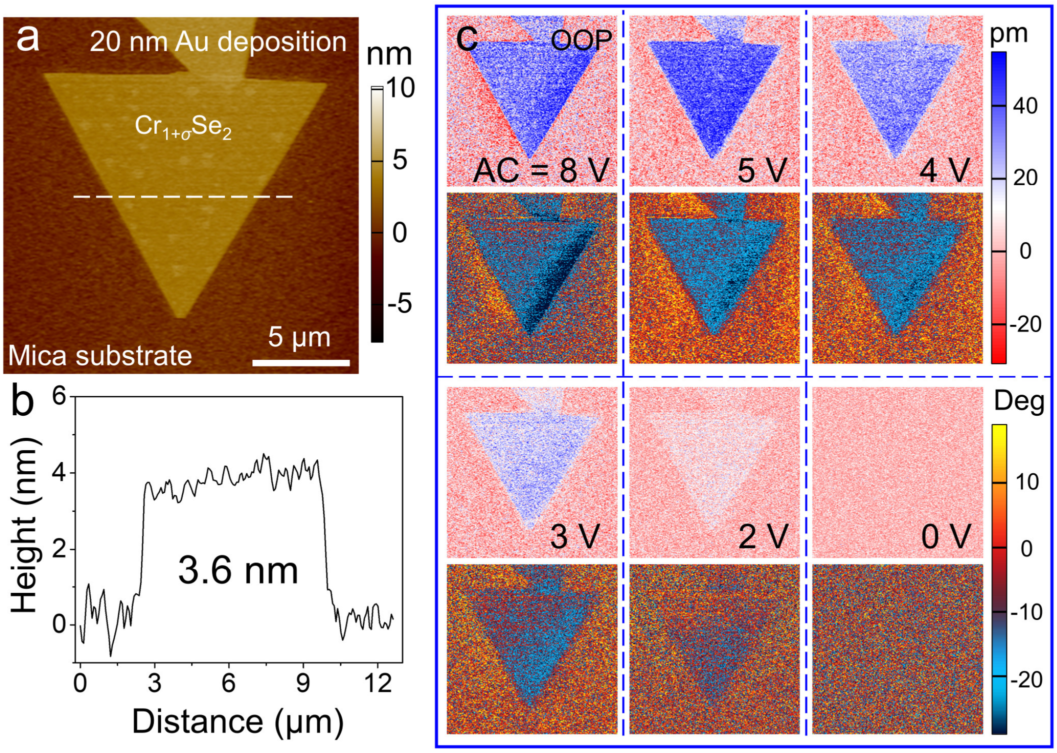

Supplementary Figure 4. PFM characterizations of an Au-coated 2D $Cr_{1+\sigma}Se_2$ nanoflake on the mica. (a) Atomic force microscopy (AFM) image, (b) height profile, and (c) OOP piezoresponse force microscopy (PFM) mapping images of a nonstoichiometric $Cr_{1+\sigma}Se_2$ triangular nanoflake on the mica substrate after the deposition of 20 nm thick Au film. Au deposition can weaken the charging and electrostatic effects, increase the uniformity of the applied electric field and avoid the flexoelectric effect. It is indicated that the surface morphology of the sample becomes rougher after Au deposition, while the piezoelectric effect is still apparent, confirming the real piezoelectricity of the nanoflakes.

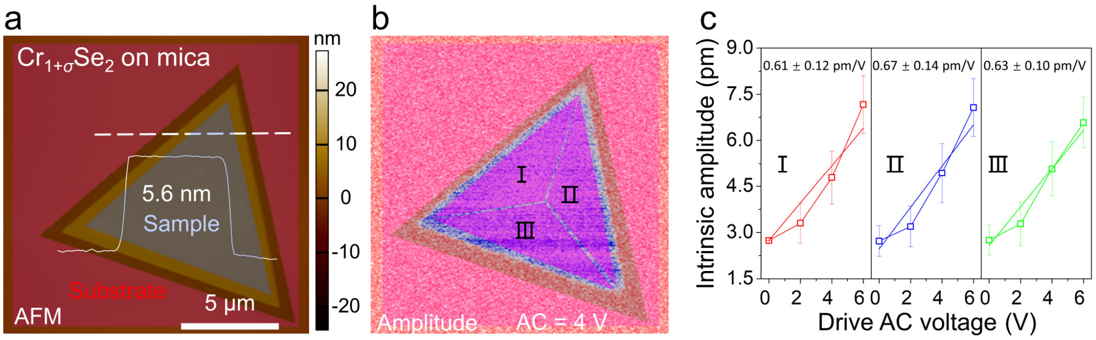

Supplementary Figure 5. Regional effective d_{33} calculation of a nonstoichiometric 2D $Cr_{1+\sigma}Se_2$ nanoflake. (a) AFM height image and corresponding profile curve of a nonstoichiometric 2D $Cr_{1+\sigma}Se_2$ triangular nanoflake. (b) Corresponding amplitude image of the nanoflake in (a) driven by an AC voltage of 4 V. Red and blue (purple) shadow masks in (a) and (b) represent regions that are used to extract substrate and sample amplitude statistic diagram for the calculation of effective d_{33} calculation. (c) The intrinsic amplitudes collected from valid areas of I, II and III as a function of the drive AC voltage curve. Error bar indicates standard deviation.

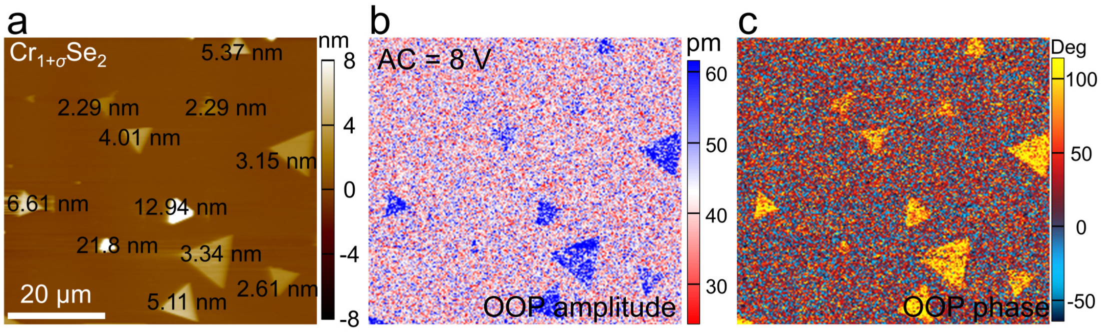

Supplementary Figure 6. Large-area PFM characterizations of nonstoichiometric 2D $Cr_{1+\sigma}Se_2$ nanoflakes. (a-c) AFM image (a), amplitude image (b) and phase image (c) of a large area nonstoichiometric 2D $Cr_{1+\sigma}Se_2$ nanoflakes with different thicknesses. It can be observed that nanoflakes with different thicknesses have different OOP amplitude intensities. Since the resonance frequency and quality factor are consistent, the piezoelectric amplitudes of different nanoflakes can be compared to evaluate thickness-dependent piezoelectricity.

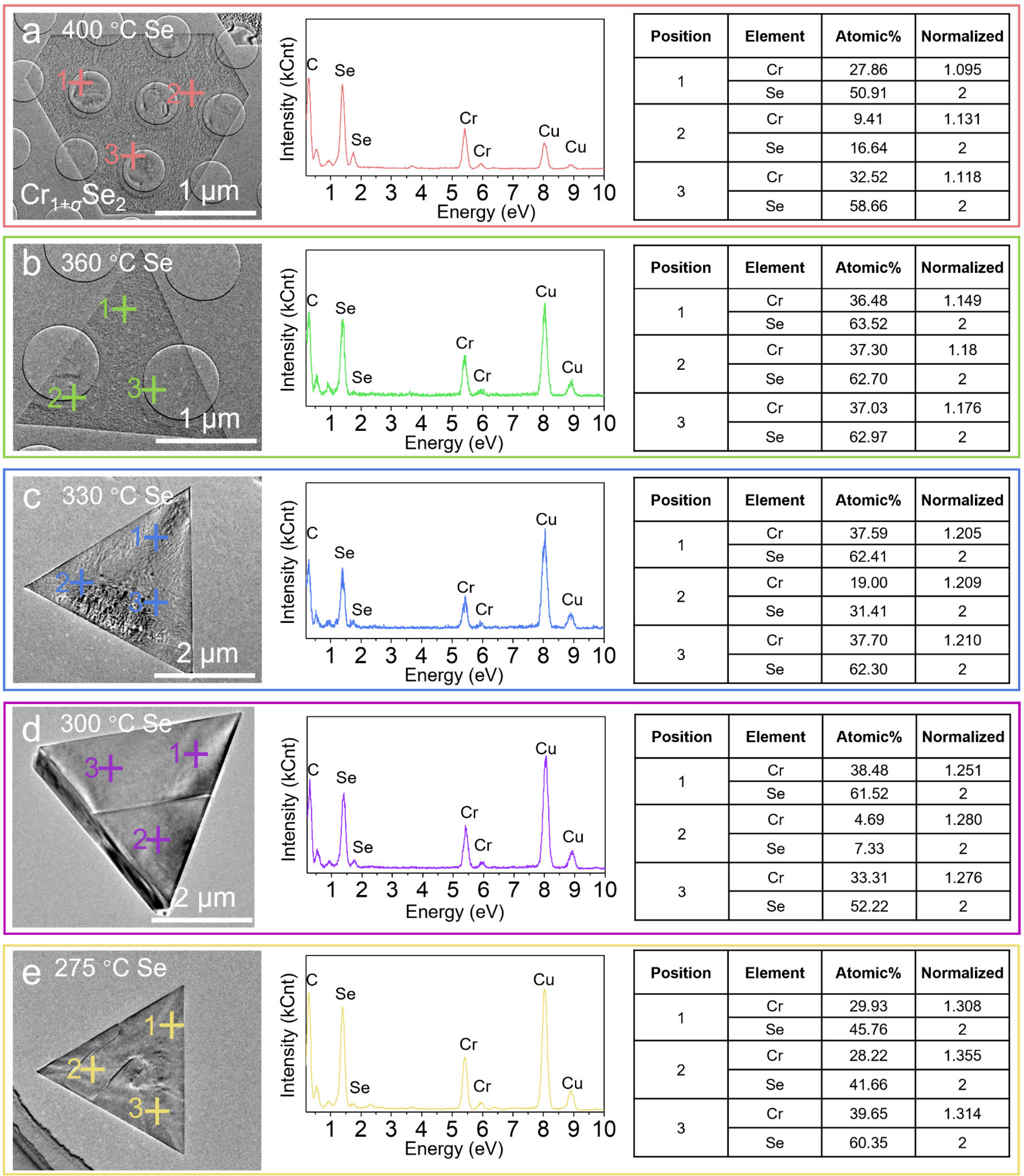

Position	Element	Atomic%	Normalized
1	Cr	27.86	1.095
	Se	50.91	2
2	Cr	9.41	1.131
	Se	16.64	2
3	Cr	32.52	1.118
	Se	58.66	2

Position	Element	Atomic%	Normalized
1	Cr	36.48	1.149
	Se	63.52	2
2	Cr	37.30	1.18
	Se	62.70	2
3	Cr	37.03	1.176
	Se	62.97	2

Position	Element	Atomic%	Normalized
1	Cr	37.59	1.205
	Se	62.41	2
2	Cr	19.00	1.209
	Se	31.41	2
3	Cr	37.70	1.210
	Se	62.30	2

Position	Element	Atomic%	Normalized
1	Cr	38.48	1.251
	Se	61.52	2
2	Cr	4.69	1.280
	Se	7.33	2
3	Cr	33.31	1.276
	Se	52.22	2

Position	Element	Atomic%	Normalized
1	Cr	29.93	1.308
	Se	45.76	2
2	Cr	28.22	1.355
	Se	41.66	2
3	Cr	39.65	1.314
	Se	60.35	2

Supplementary Figure 7. Determining chemical ratios of nonstoichiometric 2D $Cr_{1+\sigma}Se_2$ nanoflakes. (a-e) Transmission electron microscopy (TEM) image (left), X-ray energy dispersive spectroscopy (EDS) spectrum (middle), and summary of chemical ratio at three marks (right) of nonstoichiometric 2D $Cr_{1+\sigma}Se_2$ nanoflakes synthesized at Se temperature of (a) 400 °C, (b) 360 °C, (c) 330 °C and (d) 300 °C. Three points are taken on each nanoflake to measure its composition. The composition of the three points is then averaged to determine the composition of the nanoflakes prepared at different temperatures. Due to the ultrathin feature of 2D materials, the σ value determined by EDS shows a slight deviation in the second effective digit of the same nanoflake. However, it remains capable of reflecting the evolution of piezoelectricity through discussions of metal atom intercalation and qualitative analysis.

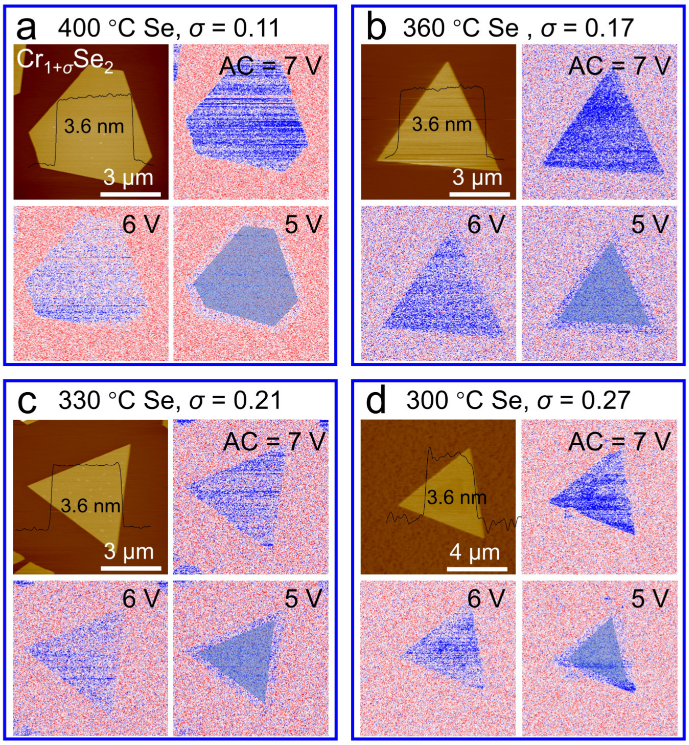

Supplementary Figure 8. PFM characterizations of nonstoichiometric 2D $Cr_{1+\sigma}Se_2$ nanoflakes with different chemical ratios. (a-d) AFM images and profiles and corresponding PFM images of nonstoichiometric 2D $Cr_{1+\sigma}Se_2$ nanoflake prepared at Se temperature of (a) 400 °C, (b) 360 °C, (c) 330 °C and (d) 300 °C. Since the AC voltage variation is identical, the change of the effective d_{33} can be qualitatively recognized by the evolution of the amplitude. The calculation of the effective d_{33} is as same as nanoflakes in Figure 3a,b. The intrinsic amplitude images are first calculated by the SHO calculation. Then, the function curves of the statistic intrinsic amplitude value with the drive voltage were made to calculate the effective d_{33} after linear fitting.

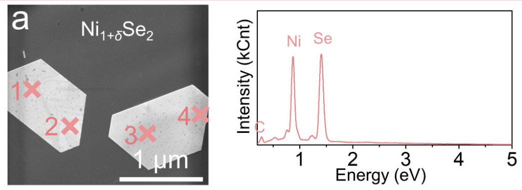

Position	1		2		3		4	
	Atomic %	Normalized	Atomic %	Normalized	Atomic %	Normalized	Atomic %	Normalized
Ni	45.4	1.66	45.5	1.67	45.3	1.66	45.0	1.64
Se	54.6	2	54.5	2	54.7	2	55.0	2

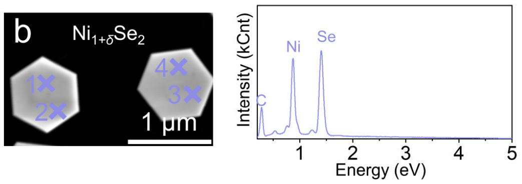

Position	1		2		3		4	
	Atomic %	Normalized	Atomic %	Normalized	Atomic %	Normalized	Atomic %	Normalized
Ni	44.1	1.5	44.4	1.6	44.3	1.59	44.6	1.61
Se	55.9	2	55.6	2	55.7	2	55.4	2

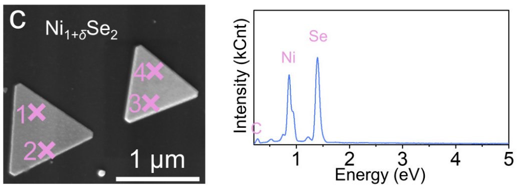

Position	1		2		3		4	
	Atomic %	Normalized	Atomic %	Normalized	Atomic %	Normalized	Atomic %	Normalized
Ni	37.7	1.21	37.9	1.22	37.3	1.19	37.4	1.195
Se	62.3	2	62.1	2	62.7	2	62.6	2

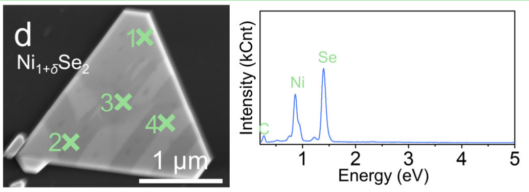

Position	1		2		3		4	
	Atomic %	Normalized	Atomic %	Normalized	Atomic %	Normalized	Atomic %	Normalized
Ni	36.0	1.125	36.7	1.16	35.7	1.11	36.2	1.13
Se	64.0	2	63.3	2	64.3	2	63.8	2

Supplementary Figure 9. Determining chemical ratios of nonstoichiometric 2D $Ni_{1+\delta}Se_2$ nanoflakes. (a-d) Scanning electron microscopy (SEM) images (upper left), EDS spectra (upper right) and chemical element ratio of four points marked in SEM image (bottom) of nonstoichiometric $Ni_{1+\delta}Se_2$ nanoflakes prepared under different chalcogen vapor pressures.

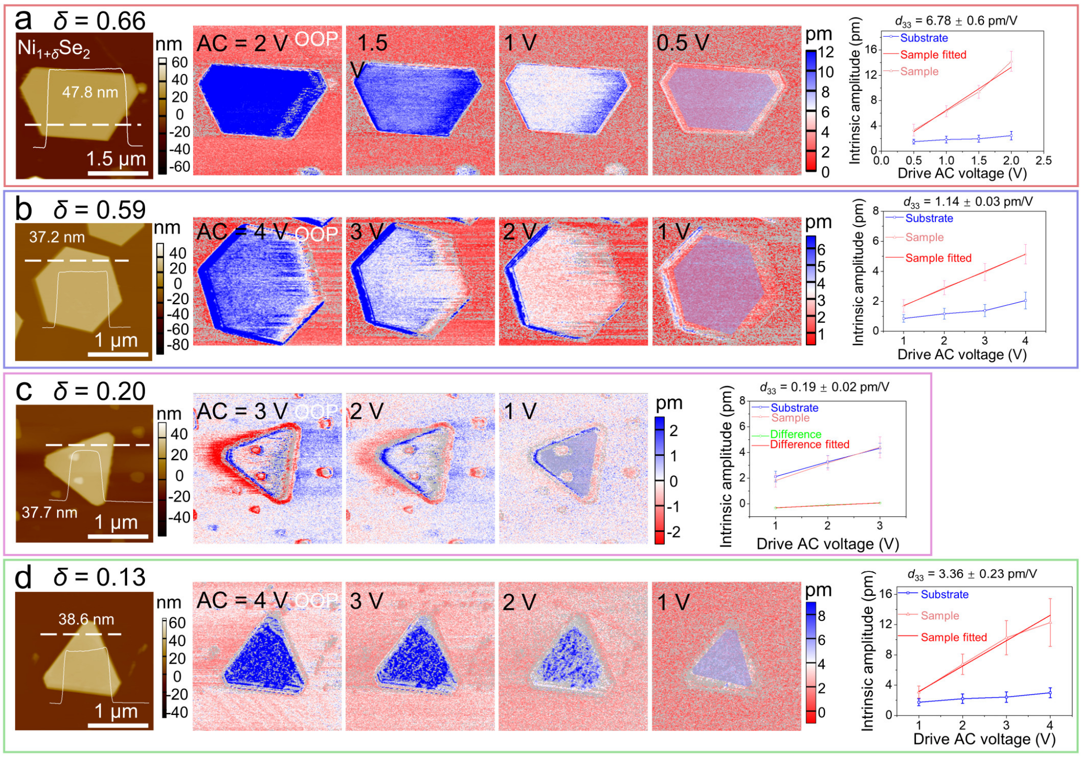

Supplementary Figure 10. PFM characterizations of 2D $Ni_{1+\delta}Se_2$ nanoflakes with different chemical ratios. (a-d) Height images (left), different drive AC voltages driven corresponding OOP intrinsic amplitude images (middle) and plots of OOP intrinsic sample amplitude (pink lines)/substrate amplitude (blue lines) as a function of drive AC voltage (right) of nonstoichiometric $Ni_{1+\delta}Se_2$ nanoflakes with different δ values. The fitted lines of intrinsic sample amplitude are marked as red color (right panel in (a), (b) and (d)). The difference (green line) in (c) is calculated by subtracting the intrinsic amplitude of the substrate from the intrinsic amplitude of the sample. The red line in (c) is the fitting line of the difference to the drive AC voltage. The δ values were determined by averaging the chemical ratio of four selected points in the SEM image (Supplementary Figure 9). According to the thickness-dependent piezoelectric coefficient evolution of nonstoichiometric $Cr_{1+\sigma}Se_2$ nanoflakes (Figure 3c), the piezoelectric coefficient is saturated when the thickness is higher than 20 nm. Thus, despite the slight difference in thickness, the piezoelectric coefficient of nonstoichiometric $Ni_{1+\delta}Se_2$ nanoflakes should be saturated for such thick nanoflakes, and the comparison is reasonable. The valid areas for counting sample amplitude were marked as light blue patterns in the last amplitude image. The effective d_{33} of each nanoflake with different δ values was calculated by fitting OOP intrinsic amplitude as a function of drive AC voltage. All error bar indicates standard deviation.

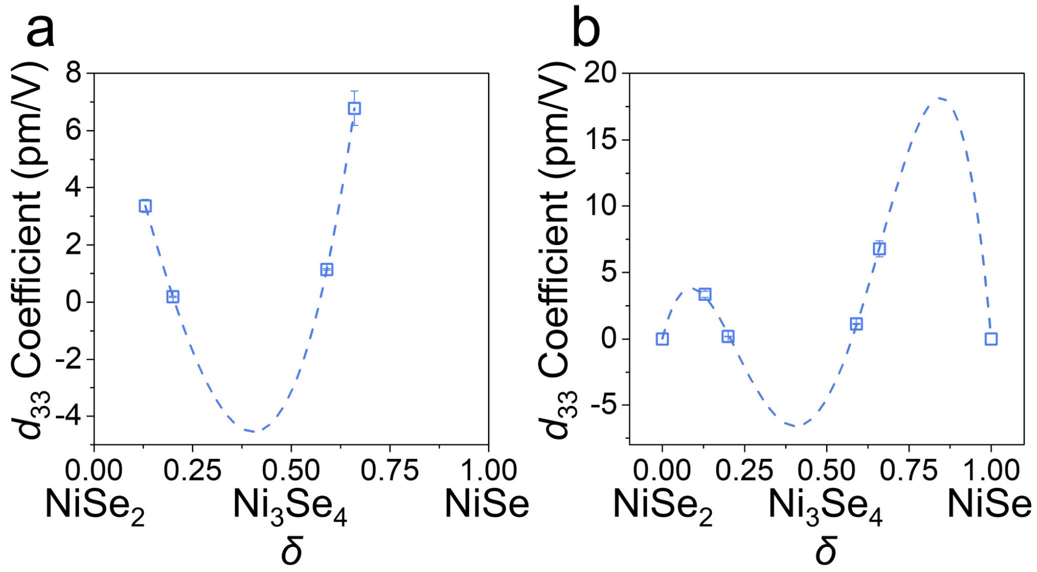

Supplementary Figure 11. Evolution of effective d_{33} as a function of δ values in nonstoichiometric $Ni_{1+\delta}Se_2$ nanoflakes. (a-b) The effective d_{33} of nonstoichiometric $Ni_{1+\delta}Se_2$ nanoflakes as a function of δ value before (a) and after (b) considering centrosymmetric $NiSe_2$ and NiSe. The scatters were fitted via polynomial fitting. All error bars indicate standard deviation.

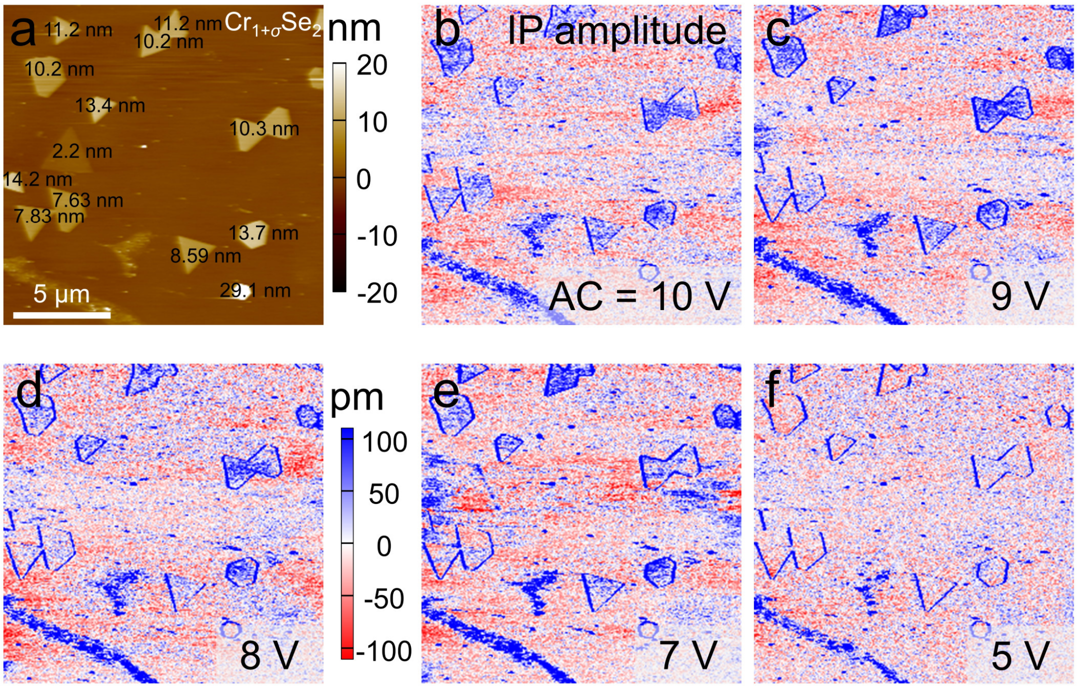

Supplementary Figure 12. IP PFM characterizations of nonstoichiometric 2D $Cr_{1+\sigma}Se_2$ nanoflakes. (a) AFM image and (b-f) IP amplitude images of nonstoichiometric 2D $Cr_{1+\sigma}Se_2$ nanoflakes at different drive AC voltages. The thickness of each nanoflake is marked. The amplitude evolution between each adjacent two images is not significant, but the piezoelectric effect is detectable over the entire drive voltage range.

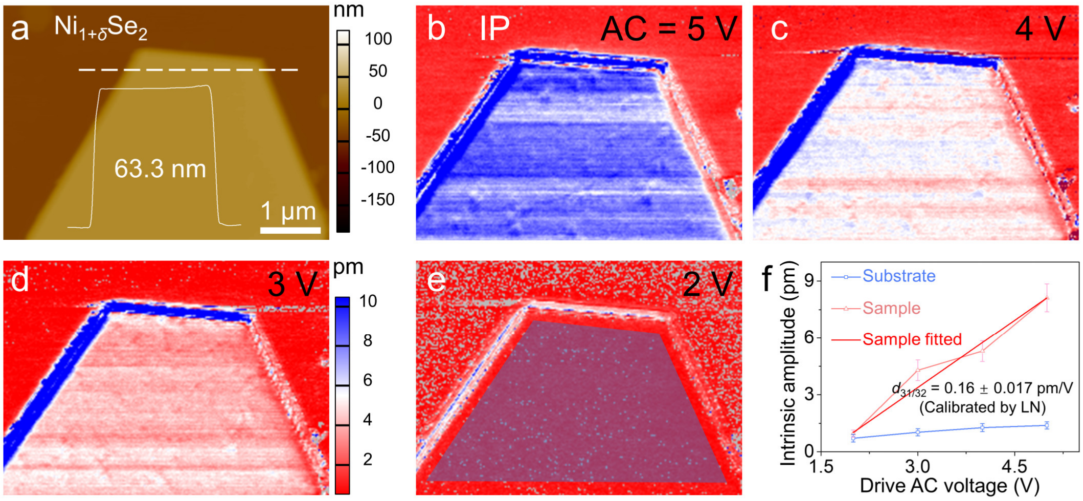

Supplementary Figure 13. IP PFM characterizations of nonstoichiometric $Ni_{1+\delta}Se_2$ nanoflakes. (a) Height image of a nonstoichiometric $Ni_{1+\delta}Se_2$ nanoflake. (b-e) Corresponding IP intrinsic amplitude images under different drive AC voltages. The δ of this nanoflake is 1.66 and the thickness of the nanoflake is about 63.3 nm to ensure the saturation of the piezoelectric coefficient. (f) The IP intrinsic amplitude as a function of drive AC voltage. The blue line, pink line and red line represent the function of the intrinsic amplitude of the substrate, the sample before and after fitting to the drive AC voltage, respectively. Error bars indicate standard deviation. The IP intrinsic amplitude typically varies with the drive AC voltage, indicating the apparent IP piezoelectric response of the nonstoichiometric $Ni_{1+\delta}Se_2$ nanoflakes. The calculated effective $d_{31/32}$ is ~0.16 pm/V after calibrated by z-cut LN crystal.

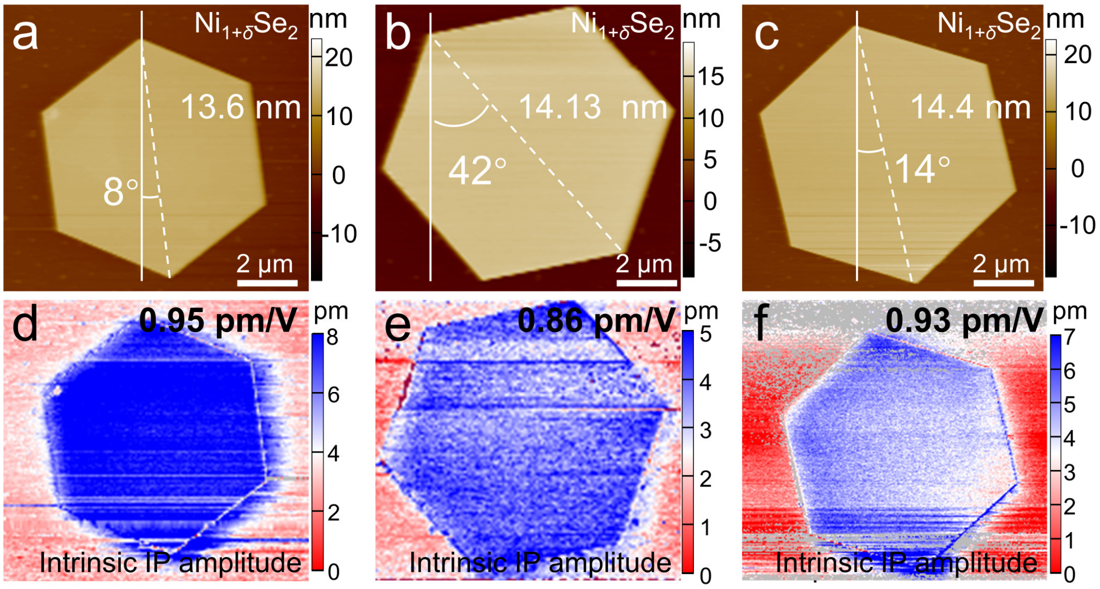

Supplementary Figure 14. IP PFM characterizations of three nonstoichiometric $Ni_{1+\delta}Se_2$ nanoflakes. (a-f) Height images (a-c) and corresponding IP intrinsic amplitude images (d-f) of three nonstoichiometric $Ni_{1+\delta}Se_2$ nanoflakes with the same thickness and chemical composition but different alignment directions. The piezoelectric coefficients in (d-f) were calculated by directly dividing the mean value of statistical amplitude to drive AC voltage. The alignment direction is uniformly defined as the degree difference between the diagonal dotted line and the vertical reference straight line, as illustrated in (a-c).

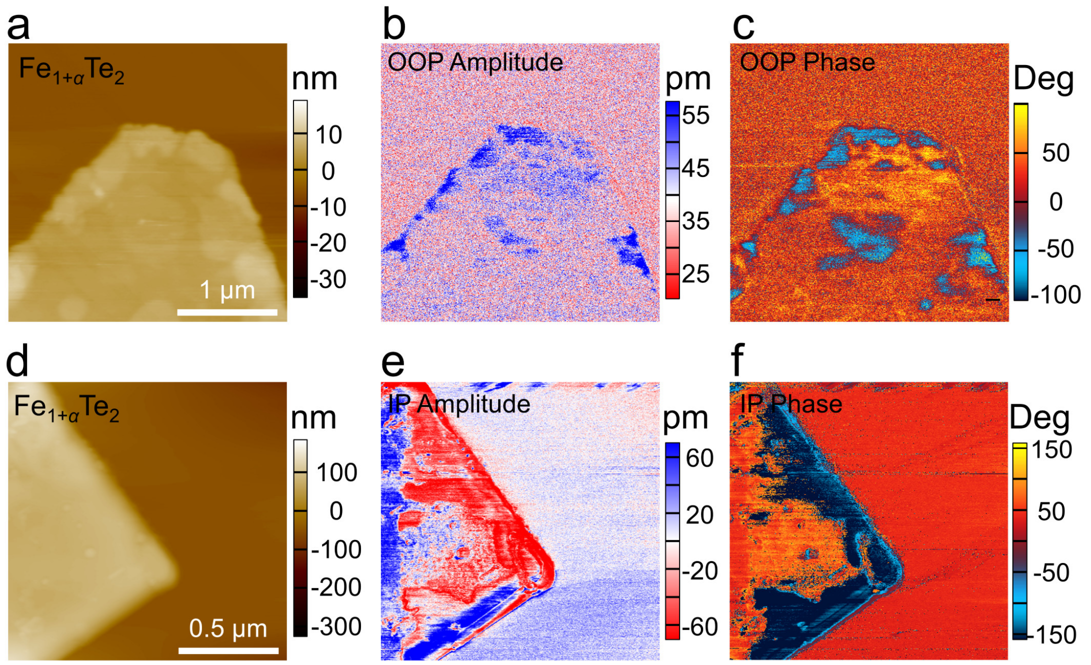

Supplementary Figure 15. PFM characterizations of nonstoichiometric $Fe_{1+\sigma}Te_2$ nanoflakes. (a-c) Magnified height image, amplitude image and phase image of a $Fe_{1+\sigma}Te_2$ nanoflake obtained by OOP PFM mode. (d-f) Height image, amplitude image and phase image of the corner of a $Fe_{1+\sigma}Te_2$ nanoflake collected by IP PFM mode. Despite the rough topography of the nanoflakes, both amplitude and phase images are significantly independent to the height images.

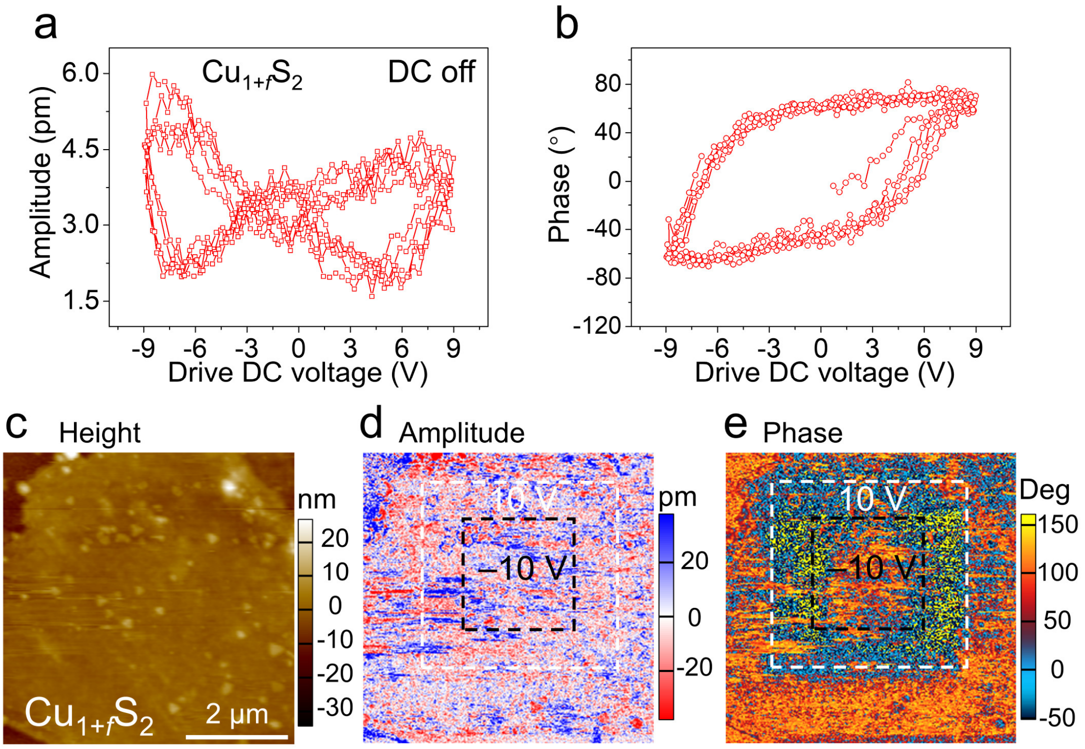

Supplementary Figure 16. PFM characterizations of a nonstoichiometric $Cu_{1+\zeta}S_2$ nanoflake. (a-b) Ferroelectric switching spectra at DC off state of a $Cu_{1+\zeta}S_2$ nanoflake. (c-e) Height image, amplitude image and phase image of a $Cu_{1+\zeta}S_2$ nanoflake after lithography of ±10 V rectangular-ambulatory-plane field (white and black dotted frames).

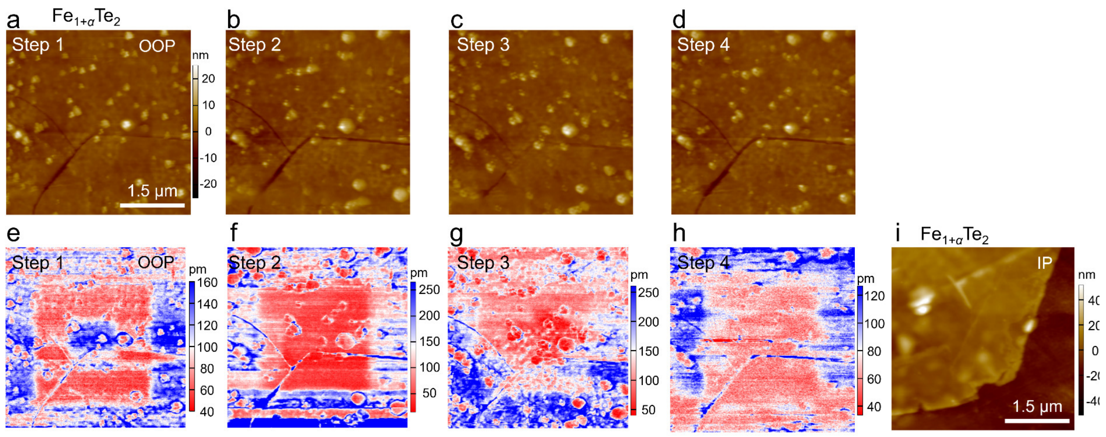

Supplementary Figure 17. Sequential lithography of a $Fe_{1+\sigma}Te_2$ nanoflake. (a-d) Hight images of a nonstoichiometric $Fe_{1+\sigma}Te_2$ nanoflake after sequential lithography with different patterns. (e-h) Corresponding amplitude images of (a-d). (i) AFM height image of a nonstoichiometric $Fe_{1+\sigma}Te_2$ nanoflake on which IP ferroelectric lithography is performed.

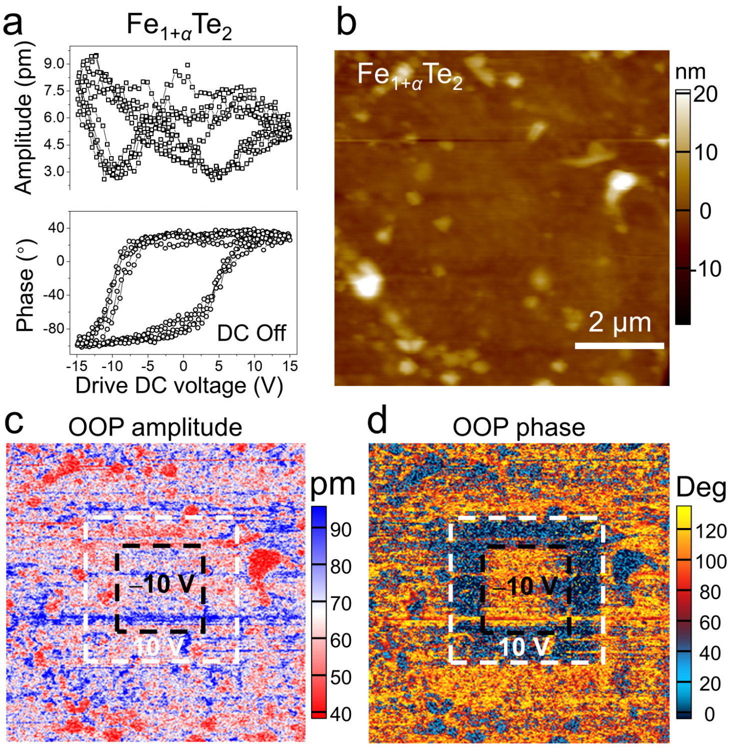

Supplementary Figure 18. PFM characterizations of a freshly prepared nonstoichiometric $Fe_{1+\sigma}Te_2$ nanoflake. (a-d) Local ferroelectric switching loops under DC off state (a), height image (b), OOP amplitude image (c) and OOP phase image (d) of a freshly prepared nonstoichiometric $Fe_{1+\sigma}Te_2$ nanoflake. The ±10 V polarized lithography was performed on the nanoflake before PFM scanning, as indicated by dotted dark frame for –10 V and dotted white frame for 10 V. The relatively high coercive voltage and phase difference of less than 180° is attributed to surface charging and electrostatics induced by insufficiently grounded.

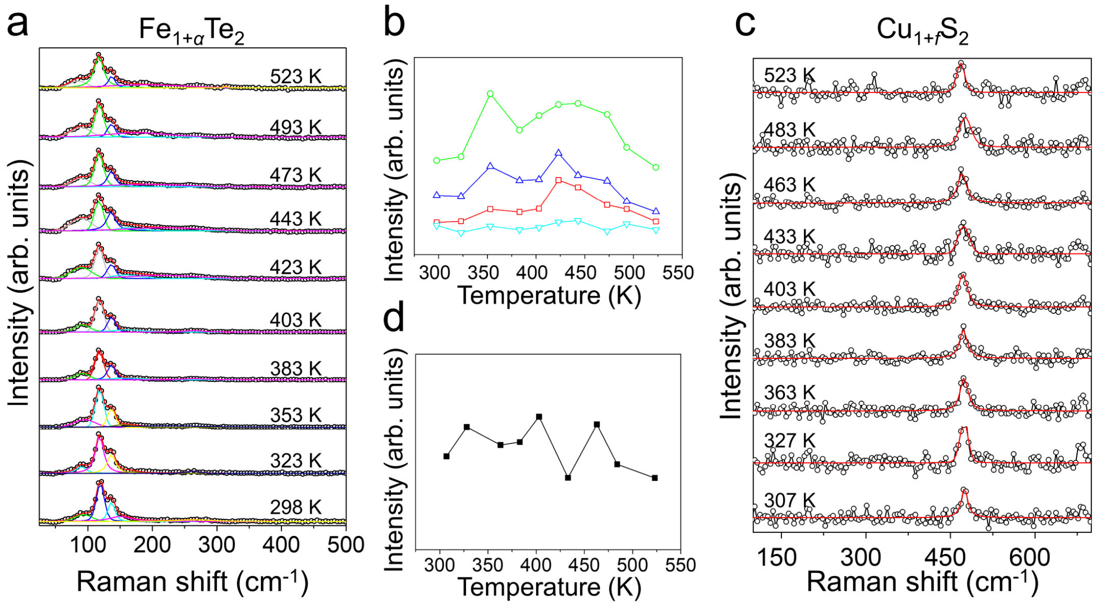

Supplementary Figure 19. Temperature-dependent Raman characterizations of nonstoichiometric $Fe_{1+\sigma}Te_2$ and $Cu_{1+\zeta}S_2$ nanoflakes. (a-d) Temperature-dependent Raman spectra and Raman peak intensity of a nonstoichiometric $Fe_{1+\sigma}Te_2$ nanoflake (a-b) and a nonstoichiometric $Cu_{1+\zeta}S_2$ nanoflake (c-d). The lines and symbols with green, blue, red and cyan colors in (b) are ascribed to peak intensity at Raman shifts of 91 cm^{-1}, 118 cm^{-1}, 137 cm^{-1} and 155 cm^{-1}, respectively. Both Raman peak intensity of $Fe_{1+\sigma}Te_2$ and $Cu_{1+\zeta}S_2$ nanoflakes present fluctuation in a small range during heating from room temperature to 523 K, indicating strong stability of ferroelectric phase with a Curie temperature above 523 K.

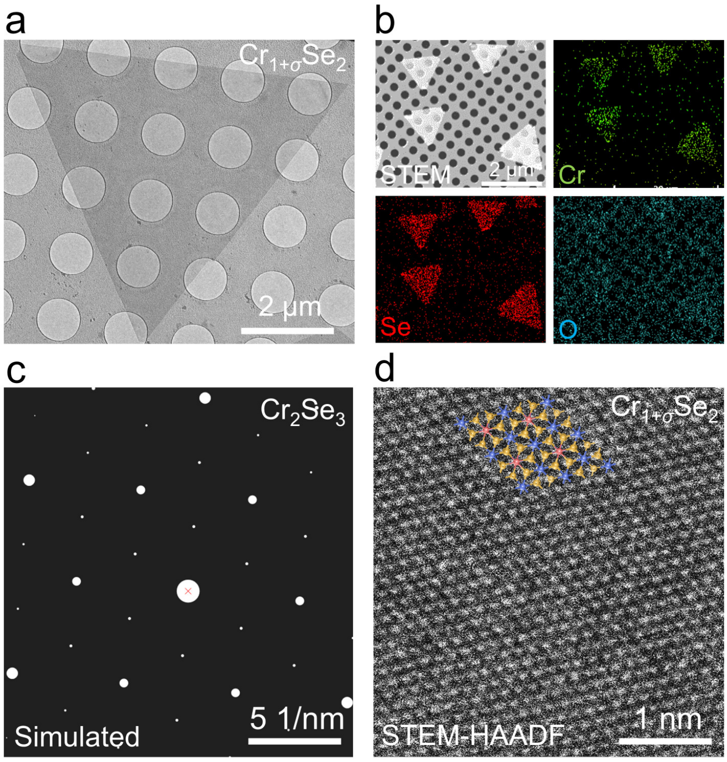

Supplementary Figure 20. TEM and EDS characterizations of nonstoichiometric $Cr_{1+\sigma}Se_2$ nanoflakes. (a) Low-magnified TEM image of a nonstoichiometric $Cr_{1+\sigma}Se_2$ nanoflake. (b) Low-magnified scanning transmission electron microscopy (STEM) image and EDS elemental mapping results (green for Cr, red for Se and cyan for O) of nonstoichiometric $Cr_{1+\sigma}Se_2$ nanoflakes. (c) Simulated selected area electron diffraction (SAED) patterns of a stoichiometric Cr_2Se_3 nanoflake. (d) Experimental high-resolution STEM high-angle annular dark-field (STEM-HAADF) image of a nonstoichiometric $Cr_{1+\sigma}Se_2$ nanoflake. The inhomogeneity of local signals in elemental mapping is due to the influence of the supporting carbon multihole film, rather than the inhomogeneity of the material.

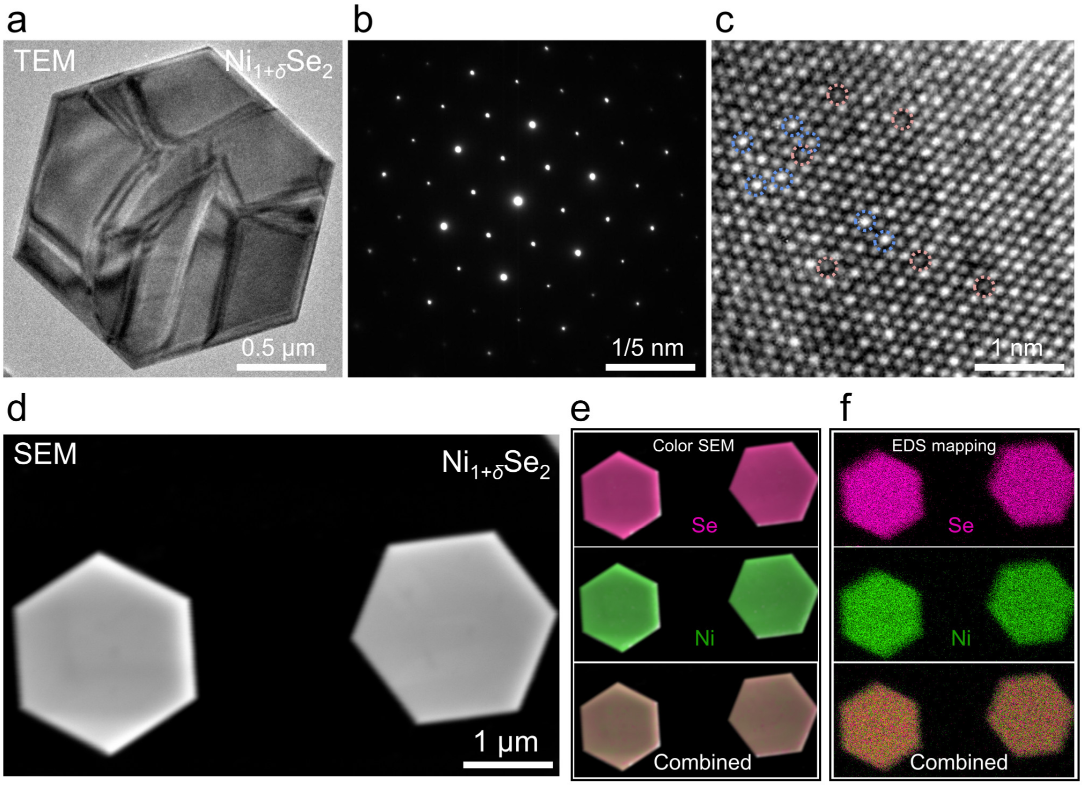

Supplementary Figure 21. TEM and EDS characterizations of nonstoichiometric $Ni_{1+\delta}Se_2$ nanoflakes. (a-c) TEM image (a), SAED patterns (b) and high resolution TEM (HRTEM) image (c) of a nonstoichiometric $Ni_{1+\delta}Se_2$ nanoflake. SAED pattern indicates a single crystalline hexagonal crystal feature. HRTEM image demonstrates some uneven interference sites, as marked by blue and red dotted circles for dark and light sites, which may be associated with defects of vacancy or extra metal atom intercalations. (d) SEM image of two nonstoichiometric $Ni_{1+\delta}Se_2$ nanoflakes. (e) False color SEM images derived from SEM contrast and EDS elemental mapping signals. (f) EDS elemental mapping images of Ni (red color), Se (green color) and integrated image of SEM and mapping images (apricot color). Both color SEM images and EDS mapping images of Se and Ni elements show a uniform distribution of signals, confirming the evenly dispersed vacancy/interstitial defects.

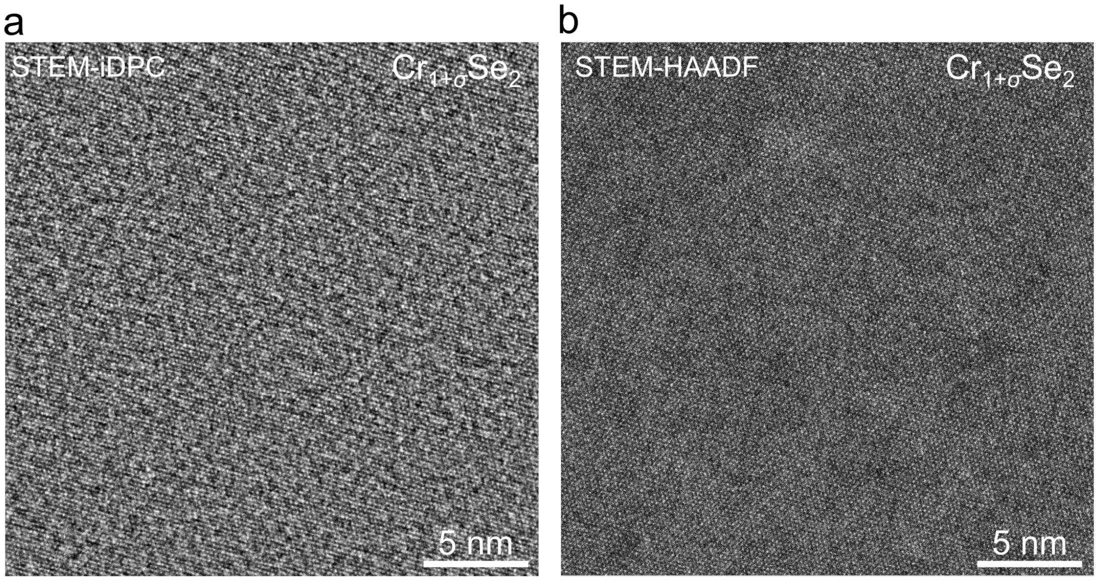

Supplementary Figure 22. STEM characterizations of nonstoichiometric 2D $Cr_{1+\sigma}Se_2$ nanoflakes. (a-b) High-magnification STEM high-angle annular dark-field (HAADF) and integrated differential phase contrast (iDPC) images with larger observation regions. The inhomogeneous defects generally exist in both STEM images.

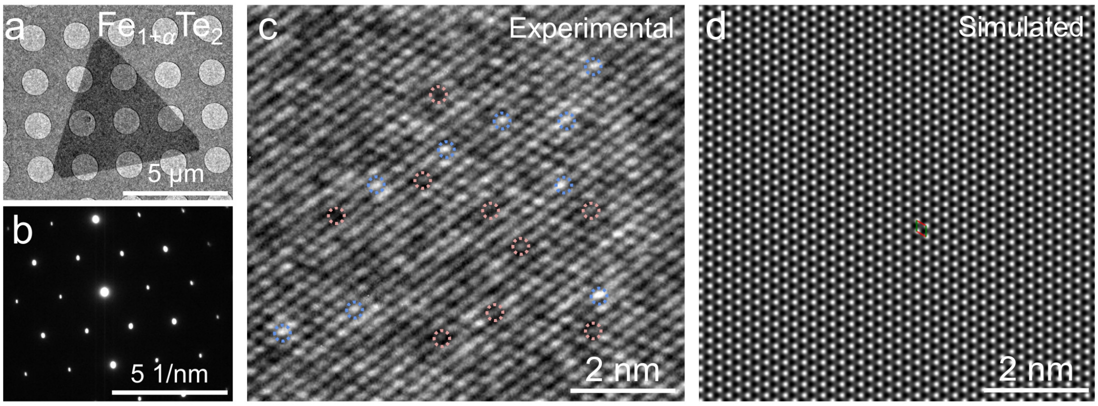

Supplementary Figure 23. TEM characterizations of a nonstoichiometric 2D $Fe_{1+\sigma}Te_2$ nanoflake. (a) Low-magnification TEM image of a triangle nonstoichiometric 2D $Fe_{1+\sigma}Te_2$ nanoflake on carbon grids. (b) Corresponding SAED pattern of a $Fe_{1+\sigma}Te_2$ nanoflake in (a). (c) Experimental HRTEM image of a $Fe_{1+\sigma}Te_2$ nanoflake in (a). Blue and red dashed circles indicate light and dark spots, respectively. (d) Simulated HRTEM image of stoichiometric $FeTe_2$ nanoflake.

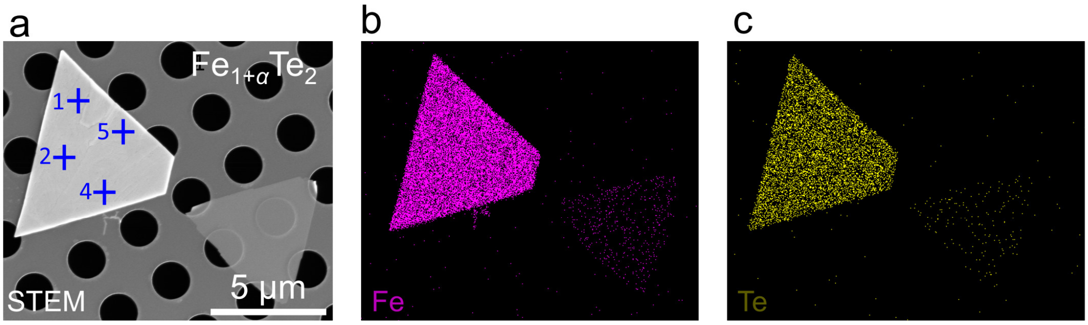

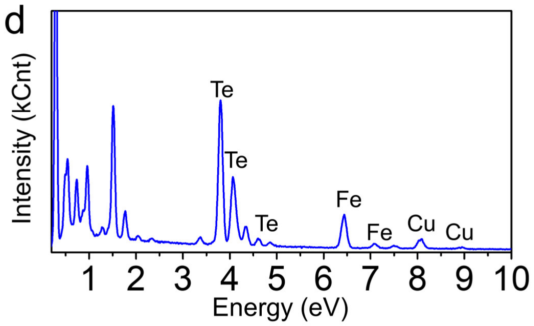

Position	Element	Atomic%	Normalized
1	Fe	37.8	1.215
	Te	62.2	2
2	Fe	37.5	1.200
	Te	62.5	2
3	Fe	38.1	1.231
	Te	61.9	2
4	Fe	37.9	1.221
	Te	62.1	2

Supplementary Figure 24. EDS characterizations of nonstoichiometric 2D $Fe_{1+\sigma}Te_2$ nanoflakes. (a-c) STEM image (a) and EDS mapping images of Fe (b) and Te (c) of nonstoichiometric 2D $Fe_{1+\sigma}Te_2$ nanoflakes. (d) EDS spectrum of point 1 and atomic composition summary of four points in nonstoichiometric 2D $Fe_{1+\sigma}Te_2$ nanoflake marked in (a).

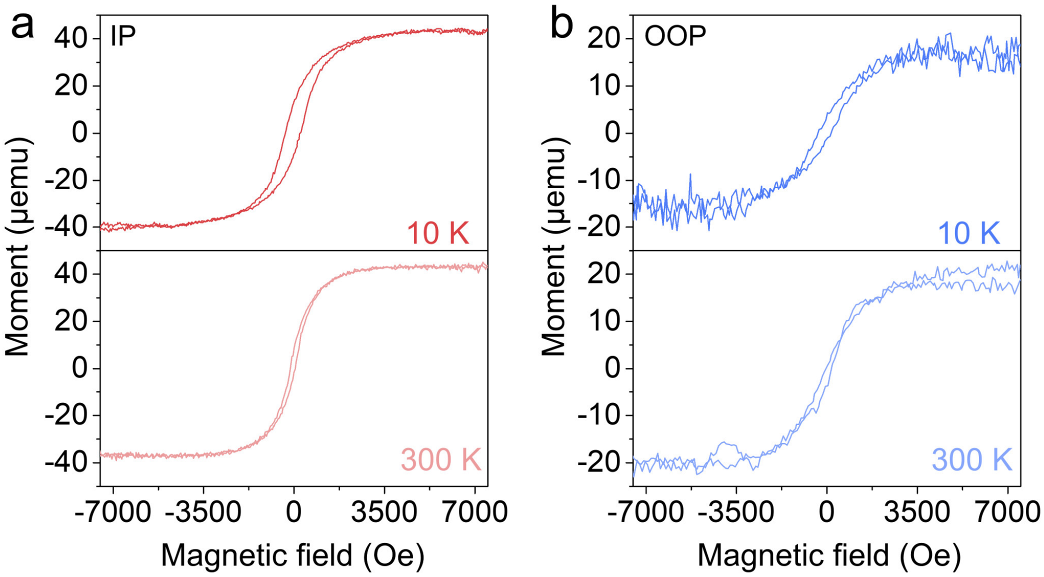

Supplementary Figure 25. Magnetic characterizations of nonstoichiometric 2D $Fe_{1+\sigma}Te_2$ nanoflakes. (a-b) *M-H* magnetization curves of nonstoichiometric 2D $Fe_{1+\sigma}Te_2$ nanoflakes under parallel (IP) (a) and vertical (OOP) (b) magnetic fields. In the IP and OOP measurement configuration, the magnetic hysteresis loops were both observed at 10 K and 300 K, indicating the long-rang and room-temperature magnetism order. The saturation magnetization and coercive field measured under IP field are higher than that under the OOP field, suggesting the easy axis direction is parallel to the van der Waals layer plane.

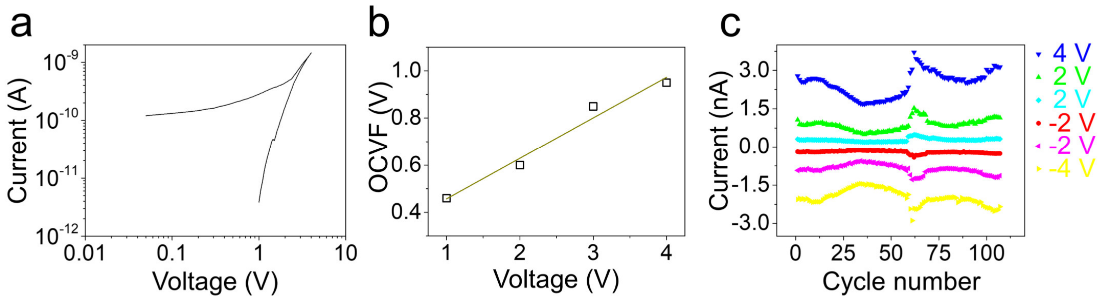

Supplementary Figure 26. Electrical transports of a nonstoichiometric 2D $Fe_{1+\sigma}Te_2$ nanoflake. (a) *I-V* curve of in logarithmic coordinates of a nonstoichiometric 2D $Fe_{1+\sigma}Te_2$ nanoflake based two-terminal devices. (b) Open circuit voltage field (OCVF) at different sweeping voltage ranges. (c) Extract current retention curves at different voltages for 109 cycles. Blue, green, cyan, red, cyan and yellow lines were read at 4 V, 2 V, 2 V, –2 V, –2 V and –4 V, respectively.

Supplementary reference:

1. Sienko, M. Nonstoichiometric compounds. *Adv. Chem. Ser.* **139** (1963).
2. Tilley, R. J. D. *Defects in solids*. (John Wiley & Sons, 2008).
3. Wang, Y. *et al.* Piezoelectric responses of mechanically exfoliated two–dimensional SnS_2 nanosheets. *ACS Appl. Mater. Interfaces* **12**, 51662–51668 (2020).
4. Apte, A. *et al.* 2D electrets of ultrathin MoO_2 with apparent piezoelectricity. *Adv. Mater.* **32**, 2000006 (2020).
5. Apte, A. *et al.* Piezo-response in two-dimensional σ-Tellurene films. *Mater. Today* **44**, 40–47 (2021).
6. Wang, X. *et al.* Subatomic deformation driven by vertical piezoelectricity from CdS ultrathin films. *Sci. Adv.* **2**, e1600209 (2016).
7. Xue, F. *et al.* Multidirection piezoelectricity in mono- and multilayered hexagonal σ-In_2Se_3. *ACS Nano* **12**, 4976–4983 (2018).
8. Hallil, H. *et al.* Strong piezoelectricity in 3R-MoS_2 flakes. *Adv. Electron. Mater.* **8**, 2101131 (2022).
9. Kang, S. *et al.* Tunable out-of-plane piezoelectricity in thin-layered $MoTe_2$ by surface corrugation-mediated flexoelectricity. *ACS Appl. Mater. Interfaces* **10**, 27424–27431 (2018).
10. Rogée, L. *et al.* Ferroelectricity in untwisted heterobilayers of transition metal dichalcogenides. *Science* **376**, 973–978 (2022).
11. Lu, A. Y. *et al.* Janus monolayers of transition metal dichalcogenides. *Nat. Nanotechnol.* **12**, 744–749 (2017).
12. da Cunha Rodrigues, G. *et al.* Strong piezoelectricity in single-layer graphene deposited on SiO_2 grating substrates. *Nat. Commun.* **6**, 7572 (2015).
