# Supplementary material for: Extendable piezo/ferroelectricity in nonstoichiometric 2D transition metal dichalcogenides
Source: Nat Commun. 2023 Dec 20;14:8470. doi: 10.1038/s41467-023-44298-5 (PMC10733392; doi:10.1038/s41467-023-44298-5)
Supplement: Supplementary file 3 — Reporting Summary [file 41467_2023_44298_MOESM3_ESM.pdf]

Corresponding author(s): Shu Ping Lau

Lasing Reporting Summary

Nature Research wishes to improve the reproducibility of the work that we publish. This form is intended for publication with all accepted papers reporting claims of lasing and provides structure for consistency and transparency in reporting. Some list items might not apply to an individual manuscript, but all fields must be completed for clarity.

For further information on Nature Research policies, including our data availability policy, see Authors & Referees.

• Experimental design

Please check: are the following details reported in the manuscript?

1. Threshold

Plots of device output power versus pump power over a wide range of values indicating a clear threshold

☐ Yes ☒ No — No related work is referred.

2. Linewidth narrowing

Plots of spectral power density for the emission at pump powers below, around, and above the lasing threshold, indicating a clear linewidth narrowing at threshold

☐ Yes ☒ No — No related work is referred.

Resolution of the spectrometer used to make spectral measurements

☐ Yes ☒ No — *Explain why this information is not reported/not relevant.*

3. Coherent emission

Measurements of the coherence and/or polarization of the emission

☐ Yes ☒ No — *Explain why this information is not reported/not relevant.*

4. Beam spatial profile

Image and/or measurement of the spatial shape and profile of the emission, showing a well-defined beam above threshold

☐ Yes ☒ No — *Explain why this information is not reported/not relevant.*

5. Operating conditions

Description of the laser and pumping conditions
Continuous-wave, pulsed, temperature of operation

☐ Yes ☒ No — *Explain why this information is not reported/not relevant.*

Threshold values provided as density values (e.g. W cm^{-2} or J cm^{-2}) taking into account the area of the device

☐ Yes ☒ No — No related work is referred.

6. Alternative explanations

Reasoning as to why alternative explanations have been ruled out as responsible for the emission characteristics
e.g. amplified spontaneous, directional scattering; modification of fluorescence spectrum by the cavity

☐ Yes ☒ No — No related work is referred.

7. Theoretical analysis

Theoretical analysis that ensures that the experimental values measured are realistic and reasonable
e.g. laser threshold, linewidth, cavity gain-loss, efficiency

☐ Yes ☒ No — No related work requires quantifying laser power.

8. Statistics

Number of devices fabricated and tested

☐ Yes ☒ No — No related work is referred.

Statistical analysis of the device performance and lifetime (time to failure)

☐ Yes ☒ No — No related work is referred.
